# In Vitro Assessment Antiparasitic Effect of Selenium and Copper Nanoparticles on *Giardia deodenalis* Cyst

**DOI:** 10.18502/ijpa.v15i3.4206

**Published:** 2020

**Authors:** Farnaz MALEKIFARD, Mousa TAVASSOLI, Kiana VAZIRI

**Affiliations:** Department of Pathobiology, Faculty of Veterinary Medicine, Urmia University, Urmia, Iran

**Keywords:** Cyst, *Giardia deodenalis*, In vitro, Selenium nanoparticles, Copper oxide nanoparticles

## Abstract

**Background:**

Application of chemotherapy to treatment of parasitic disease of man and animals can be problematic due to different adverse effects. As a result, there is an increasing interest in nanoparticles as new therapeutic tools against these diseases. This study was designed to evaluate the effect of selenium and copper oxide nanoparticles on *Giardia deudenalis* cysts in vitro, as well as comparing it to that of metronidazole.

**Methods:**

The cysts were taken from the stools of patients in Urmia, Iran, during 2017–2018. The cysts were taken from stool and were concentrated and isolated on 0.85 M-sucrose. Then, selenium and copper oxide nanoparticles were prepared at concentrations of 0.15, 0.3, and 0.6 mg/ml. The effect of nanoparticle’s various concentrations at 10, 15, 30, 60, and 180 min were evaluated and compared to control groups. Obtained data was recorded and statistically analyzed.

**Results:**

Copper oxide nanoparticles at a concentration of 0.6 mg/ml and selenium nanoparticles at a concentration of 0.3 mg/ml had the same effect as of metronidazole in killing of *Giardia* cysts. The cytotoxic effects of selenium and copper oxide nanoparticles, compared with metronidazole, on *Giardia* cysts, showed an increase of fatality rate due to extend exposure time and nanoparticle’s concentration (*P*<0.05).

**Conclusion:**

Selenium and copper oxide nanoparticles are as efficient as metronidazole, for killing *Giardia* cysts in vitro.

## Introduction

Giardiasis is one of the important parasitic disease that results a significant health problem all over the world. G. *deudenalis* is the most common human intestinal protozoa especially in temperate regions. It also exists in animals such as birds, amphibians, rodents, and some mammals (1). This protozoa can cause several anomalies like malnutrition, nausea, diarrhea, cramps, jaundice, avitaminosis, and weigh loss in children (2).

Metronidazole (Flagyl) is the most common drug used to cure giardiasis and it is one of the derivatives of nitroimidazole and is able to kill anaerobic bacteria and protozoa. Furazolidone and quinacrine are among the other drugs prescribed to cure giardiasis (3,4). Metronidazole has long been used to cure protozoan, bacterial and fungal infections, however there are some recent reports regarding carcinogenic and tetratogenic effects of metronidazole on laboratory rats. Reports also indicate the drug resistance of some of *Giardia* isolates. Several side-effects such as headache, nausea, dizziness and the drug’s metallic taste has been reported by metronidazole users (3,5).

Due to their powerful effect (cytotoxic and inhibitory effect) and less harm, nanoparticles are better replacements for removing parasites and the vaccines produced by nanoparticles are effective in preventing and controlling the parasites (6). Tanoparticles of silver, selenium, chitosan, and oxidized metals have growth inhibitory or cytotoxic effect on various parasites such as *Giardia*, *Leishmania*, malaria, *Toxoplasma*, and insect larva (6).

Selenium (Se) is a trace element with various positive effects on human health and can prevent cancer and it has antiviral, anti-inflammatory and antioxidant effects (7). Recent improvements in medicine has led to fabrication of nanosize selenium. Various in vivo and in vitro studies investigated the effects of selenium nanoparticles and different forms of selenium (8–15).

Nanoparticles (NPs) of copper oxide (CuO) have several different applications and they are used in various areas such as catalysts, batteries, magnetic storage media, solar energy and superconductors (16,17). These nanoparticles have antibacterial activity against *Klebsiella pneumoniae*, *Pseudomonas aeruginosa*, *Salmonella paratyphi* and *Shigella* strains (18).

As far as we know, none of the researches have analyzed the antigiardial effects of copper oxide and selenium nanoparticles. To this end, we investigated the effects of these nanoparticles on *G. deudenalis* cysts in vitro as an alternative to metronidazole in order to obliterate the side-effects caused by metronidazole. Besides, we compared the effects of metronidazole and selenium and copper oxide nano-particles on *G. deudenalis* cysts in vitro.

## Materials and Methods

### Nanoparticles

The cysts were taken from the stools of patients in Urmia, Iran, during 2017–2018. The nanoparticle powders of selenium and copper oxide with average sizes of 10 and 10–45 nanometers (nm), respectively were obtained from US Research Nanomaterials, USA. They were dispersed in ultrapure water and were sonicated at 100W and 40 kHz for 40 min in order to obtain a homogenous suspension. The NPs were then serially diluted in sterile ultrapure water and additionally sonicated for 40 min. While dilution, small magnetic bars were put in the suspensions so that the particles are not aggregated or deposited.

### Preparation of G. duodenalis cysts

**Fig. 1:**
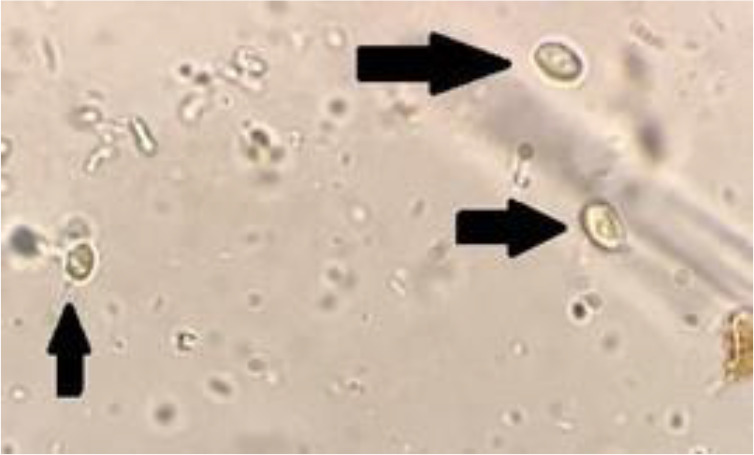
purified *G. duodenalis* cysts(×400)

Sucrose density gradient was used to isolate *G. duodenalis* cysts. Initially, 10–15 mL of distilled water was added to the stool samples which contained desired number of cysts and the suspension was prepared. After that, a layer of wet gauze was utilized to refine the suspension. The suspensions were poured into tubes and they were centrifuged for 5 min. The upper layers of the suspensions were collected and set aside. This process was repeated for 3 times. Then, 3 mL 0.85 sucrose solution and afterwards 3 mL of the prepared stool suspension were added to the tubes. In the next stage, the tubes were placed in a refrigerator at 4°C and were centrifuged for 10 min. The result was a four-layered structure in which the *G. duodenalis* cysts were in the shape of cloud rings and were in the middle layer. A Pasteur pipette was used to take the cloud layers and these layers were transferred to another tube. Afterwards, 3–5 mL distilled water was added to the samples and the tubes were centrifuged for 5 minutes at 600 g for the isolation of sucrose solution. The layer formed on the top of the tubes were set aside and the sedimentations were centrifuged for 5 min at 600 g. This process was repeated for two times. Finally, the upper layer was set aside and the sedimentation had the purified cysts (Fig. 1) (19).

### In vitro assay

**Fig. 2:**
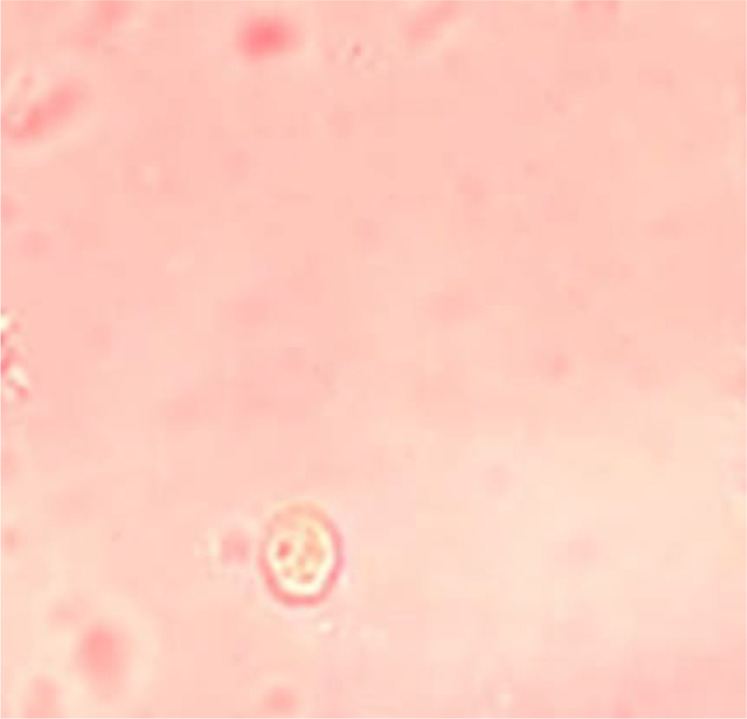
*G. duodenalis* cysts after exposure NPs and staining with 0.1%

In order to assess the effects of selenium and copper oxide nanoparticles in killing *G. duodenalis* cysts, three different concentrations (0.15, 0.3 and 0.6 mg/ml) were used with various exposure times (10, 15, 30, 60, and 180 min). To this end, the purified cyst samples (500 μl containing 5000 cyst) were exposed to 500 μl of nanoparticles at different concentrations (0.15, 0.30, and 0.6 mg/ml). Metronidazole at a concentration of 5 mg/ml was also used for 10, 15, 30, 60 and 180 minutes (20). 0.1% eosin vital staining and hemocytometer along with an optical microscope were used to analyze the bioavailability of *G. duodenalis* cysts (Fig. 2). All processes were triplicated and the following formula was used to calculate the percentage of the dead cysts: (D/( L + D ))× 100, L is the number of living cysts, D is the number of dead cysts (21).

### Statistical Analysis

Statistical analysis was conducted by SPSS software (ver.19, Chicago, IL, USA). ANOVA was used to assess the difference between test and control groups. Values less than 0.05 (*P*<0.05) were considered significant.

## Results

**Fig. 3:**
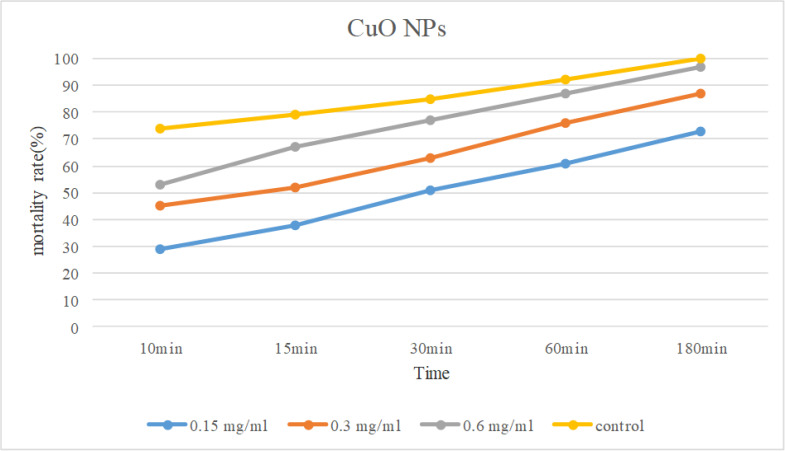
Comparison of mortality rate of CuO NPs in diferent concentration and expoture time

The antigirdial effects of different concentrations of nanoparticles of CuO and Se (0.15, 0.3 and 0.6 mg / ml) in different durations (10, 15, 30, 60, and 180 minutes) are shown in Table 1 and 2. The average results of three tests on the effect of CuO nanoparticles at different concentrations and durations on *G. duodenalis* cysts showed that the addition of nanoparticles with varying concentrations led to a significant reduction of alive cysts and the highest reduction was related to the concentration of 0.6 mg/ml in 180 min. With increasing exposure time for each concentration, the mortality rate in *G. duodenalis* cysts increased at concentrations of 0.15, 0.3 and 0.6 mg/mL(P<0.05), so that mortality rate after 10 min was derived 29%, 44%, and 53%, respectively, while it increased to 73%, 87%, and 97%, respectively, after 180 min (Fig. 3).

**Table 1:** Mortality rates(%) at different exposure time of treated *G. duodenalis* cysts by different concentrations of CuO NPs

***180 ( % )***	***60 ( % )***	***30 ( % )***	***15 ( % )***	***10 min ( % )***	***CuO NPs concentration (mg/ml)***
73	61	51	38	29	0.15
87	76	63	52	44	0.3
97	87	77	67	53	0.6
100	92	85	79	74	Control(Metronidazole)

**Table 2:** Mortality rates at different exposure time of treated *G. duodenalis* cysts by at different concentrations of Se NPs

***180 ( % )***	***60 ( % )***	***30 ( % )***	***15 ( % )***	***10 min ( % )***	***Se NPs concentrations (mg/ml)***
77	64	53	42	34	0.15
96	81	73	61	49	0.3
100	91	83	77	70	0.6
100	92	85	79	74	Control(Metronidazole)

**Fig. 4:**
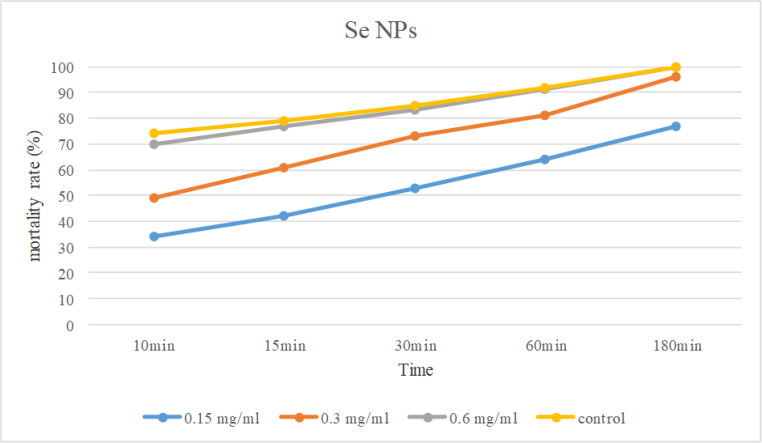
Comparison of mortality rate of Se NPs in diferent concentration and expoture time

The exposure duration of 180 minutes at a concentration of 0.6 mg/ml to selenium nanoparticles killed all the cysts. Increasing the exposure time in different concentrations led to significant increase in mortality rate (*P*<0.05) (Fig. 4).

Se and CuO nanoparticles at three concentrations on *G. duodenalis* cysts at different intervals, compared with metronidazole on *Giardia* cysts increased mortality rate due to extended exposure time and increase in nanoparticle’s concentration (*P*<0.05).

## Discussion

Nanoparticles has their own unique physicochemical properties and they are tiny in size and have great surface area and electrical charge and special shape (22). Nanoparticles of metal oxide are used in different scientific fields (23) and they are of great importance in drug delivery and treatment of various cancers (24). Due to their large surface-volume ratio, nanoparticles can interact with various biological molecules and microorganisms and are able to disturb negative activity of various parasites. They can also more frequently enter the cell compared to other particles (25). During the recent years, nanoparticles (NPs) have received more attention as antiparasitic agents (26). Nanoparticles can kill various protozoa such as *Entamoeba histolytica* and *Cryptosporidium parvum* (^26^), *Leishmania infantum* (27) and *Giardia* (20). Our study analyzed the antigiardial effects of CuO and Se nanoparticles in vitro. To the best of our knowledge, this is the first study to evaluate the in vitro effects of CuO and Se nanoparticles on *G. deudenalis* cysts.

This study demonstrated that *G. deudenalis* cysts had the highest fatality rate when exposed to CuO nanoparticles for 180 minutes. The nanoparticles of CuO were effective in killing *E. histolytica* and *C. parvum* cysts when exposed for 180 minutes in vitro (26). The nanoparticles of CuO have antibacterial properties. Cu nanoparticles had inhibitory effects on *Escherichia coli*, *Klebsiella pneumoniae*, *Pseudomonas aeruginosa*, *Propionibacterium acnes* and *Salmonella typhi* (18). The nanoparticles of copper have antibacterial properties and could act on various bacteria (28). Copper interacts with *sulfhydryl* groups(–SH) and leads to denaturation of protein. These nanoparticles are also able to affect cell membrane and it is due to the relation of amines and carboxyl groups on organism’s cell surface as *Bacillus subtilis* bacteria (29,30). These nanoparticles bind with DNA molecules within the cell and they change the helical structure between nucleic acid strands (31). The ions of Copper were able to enter the bacterial cells and disturb the biochemical processes (32).

This study showed that all of the *Giardia* cysts were killed after 180 min of exposure to selenium nanoparticles (0.6 mg/ml). The nanoparticles of selenium can inhibit the growth of some bacteria such as *Staphylococcus aureus* and pathogenic *E. coli* (33,34). Furthermore, the nanoparticles of selenium significantly inhibited the growth of *E. granulosus* protoscoleces and *L. major* on in vitro and in vivo models (35, 36). The nanoparticles of selenium could act as antileishmanial agents, and different concentrations of selenium nanoparticles significantly inhibited the growth of promastigotes of sensitive and Glucantime (MA) resistance *L. tropica* in a dose-dependent manner (25).

In the present study, *G. deudenale cyst* showed gradual increase in mortality rate with the increase in Se and CuO NPs concentrations. Duration of exposure affected the mortality rate of parasitic cysts and any increase in the exposure time leads to higher mortality rate. The results of this study are in line with that of Saad et al. who showed that CuO nanoparticles were effective in treating *E. histolytica* and *C. parvum* parasites and they also noted that this process was time and dose dependent (26).

## Conclusion

CuO and Se NPs had a high fatality effect on *G. deudenale* cysts and therefore could be recommended as an effective compound and water treatment for removing *G. deudenale* cysts.
